# Sleep disruptions and the pathway to psychosis: An in‐depth case and literature review

**DOI:** 10.1002/ccr3.9108

**Published:** 2024-06-16

**Authors:** Jeffrey Kim, Sakshi Prasad, Nikhita Suzanne Roshan, Bushra Farah Hasan, Gurtej Gill, Sasidhar Gunturu

**Affiliations:** ^1^ Department of psychiatry Bronxcare Health System Bronx New York USA; ^2^ Vinnic'kij nacional'nij medicnij universitet imeni Mikoli Pirogova Vinnytsya Ukraine; ^3^ Father Muller Medical College Mangalore India; ^4^ American University of the Caribbean School of Medicine Cupecoy Sint Maarten

**Keywords:** case report, first episode psychosis, insomnia, schizophrenia

## Abstract

**Key Clinical Message:**

The case highlights an unusual presentation where sleep issues preceded psychotic symptoms, implying link between disrupted sleep and psychosis onset. Earlier symptoms were viewed as depression but may have signaled psychosis exacerbated by insomnia.

**Abstract:**

Sleep disorders, prevalent yet frequently overlooked in individuals with psychotic disorders, have significant associations with the onset and severity of psychosis. Here we describe the case of a patient who first presented with insomnia, but whose condition improved with the use of risperidone and was diagnosed with first‐episode psychosis. Multiple studies emphasize the critical relationship between sleep disturbances and psychosis, particularly in the lead‐up to first‐episode psychosis. Structural abnormalities in the brain, notably the thalamus, combined with neurotransmitter imbalances involving dopamine and acetylcholine, seem pivotal in this interrelation. The connection between dopamine, sleep disturbances, and psychosis, specifically the role of D2 dopamine receptors, highlights a potential pathway bridging sleep irregularities with psychosis. The study underscores the need for further research to delineate the relationship between sleep disturbances and psychosis and to assess the efficacy of various therapeutic interventions targeting both conditions.

## 1 INTRODUCTION

Sleep disorders are frequently observed and commonly underdiagnosed in individuals with psychotic disorders, yet the causative relationship between the two remains elusive. One study found in a sample of 120 individuals with psychosis, 34.1% also reported a diagnosis of a concomitant sleep disorder.^1^, ^2^

The presence of at least one sleep disorder was linked to heightened psychopathological symptoms, such as increased severity in paranoia, hallucinations, and disorganized thinking.^1^, ^3^ Insomnia, in particular, correlates with a heightened risk of suicidality among schizophrenia patients.^4^, ^5^ A randomized controlled trial examining sleep loss and incidence of psychotic symptoms found that sleep deprivation increased the incidence of psychotic experiences.^1^, ^3^ Disruptions to both the continuity and restorative nature of sleep have been identified as predictive risk factors for the subsequent manifestation and severity of psychotic symptoms. Specifically, difficulties with sleep initiation and maintenance (i.e., sleep discontinuity and increased latency), as well as impaired sleep quality and depth of rest obtained, are associated with the worsening of psychotic experiences on follow‐up assessments.^6^, ^7^ Preserving healthy sleep architecture and functioning appears important for mitigating the likelihood and impact of psychosis, highlighting sleep as a modifiable target in psychosis prevention and management efforts.^6^, ^7^ Significantly, individuals with concurrent psychotic disorders and insomnia demonstrated diminished sleep efficiency, as measured by the proportion of time spent in bed dedicated to actual sleep, in contrast to the controls without insomnia.^1^, ^3^

Psychotic disorders can also influence the sleep quality of affected individuals. A case‐control study by Rasmussen^8^ found that aberrations in the callosal microstructure within the white matter of ultra‐high‐risk individuals were linked to observed disruptions in sleep‐wake functioning. An ultra‐high risk (UHR) individual for psychosis refers to someone who is considered clinically at very high risk of developing a full‐blown psychotic disorder, such as schizophrenia, within the next year.^9^

While the use of antipsychotics in managing psychotic disorders frequently induces daytime drowsiness, this effect might prove beneficial for patients with concurrent sleep disorders. However, there is limited research examining pharmacological interventions targeting both conditions simultaneously.^4^

## 2 CASE HISTORY

A 20‐year‐old male was evaluated at a major tertiary hospital after he was found calling his mother while crying and speaking incoherently. He reported having trouble sleeping and feeling exhausted for more than 8 weeks. He was eventually admitted with the diagnosis of Major Depressive Disorder (MDD) and discharged on Bupropion 100 mg twice daily for depression with Trazodone 150 mg daily for insomnia. Following discharge from his initial hospitalization, the patient's psychiatric history was notable only for one additional outpatient psychiatry visit at a major community hospital. During this encounter, he received a repeat diagnosis MDD and was prescribed Bupropion 100 mg twice daily as before, with no other mental health treatment documented after that single follow‐up appointment.

The patient was readmitted to the hospital 14 days after his outpatient visit. According to the patient's mother upon readmission, his concerning episode began 2 days prior when his friend contacted him via text message. The friend reported observing the patient standing and staring fixedly at a basement window at his home. In response, the friend assisted in bringing the patient back to his mother's care and supervision. The next day, the patient was seen by the mother staring at trains going by at the subway station platform, smiling inappropriately, and occasionally yelling as if recoiling in pain and surprise. The mother activated emergency medical services (EMS) to bring him to the emergency department and noted that the patient was cooperative and himself requested medical assistance, saying, “I need to go to the hospital.”

Upon initial admission to the unit, the patient displayed signs of depression, coupled with periods of disorganization. Nursing staff frequently commented that he had a “lost” demeanor, often gazing blankly at walls and resisting engagement with healthcare providers. Bupropion 100 mg twice daily was introduced to his treatment regimen, leading to a reported increase in his activity levels. However, this medication seemed to exacerbate his other symptoms.

The patient then began to show episodes of emotional outbursts with aggression and bizarre behavior that necessitated the use of emergent medications, sometimes crying while clutching his head as if in agony, and running through the hallways. He would at times also appear internally preoccupied and laughing inappropriately as if responding to internal stimuli.

## 3 METHODS

The patient's Bupropion was discontinued in favor of oral risperidone 4 mg daily. The patient's symptoms, including his sleep, gradually improved with this single antipsychotic regimen. He appeared much more organized, calm, and pleasant, and was eventually discharged after returning to his baseline. The patient was also received comprehensive evaluation by a clinical psychology team, utilizing the Modified Mini Mental Status Exam (3MS), Wechsler Adult Intelligence Scale‐Fourth Edition (WAIS‐IV), Personality Assessment Inventory (PAI), and Rorschach, who also supported the assessment of first‐episode psychosis.

## 4 CONCLUSION AND RESULTS

The unique aspect of this case is that the patient's sleep disturbances appeared to precede and possibly contribute to the onset of his psychotic symptoms, which then improved with treatment with risperidone. This implies a robust connection between disrupted sleep and the emergence of psychotic features, which is not as frequently observed as the link between sleep issues and mood disorders. Earlier in the patient's clinical course, he exhibited symptoms such as crying and disorganized speech that were attributed to his depression at the time. However, in retrospect, it seems these could have represented an early psychotic process that was exacerbated by disturbed sleep patterns.

## 5 DISCUSSION

There is strong evidence associating sleep disturbances with psychosis, with limited literature exploring the role of sleep in first‐episode psychosis. The most frequent age range for one to experience first‐episode psychosis is between 16 and 35 years,^5^, ^10^ and it is estimated that up to 80% of these individuals may suffer a repeat episode in the next 5 years.^5^, ^11^ A link between psychotic break and sleep disturbance lies in the prodromal phase (also called “ultra‐high risk” or “at‐risk mental state”), preceding first‐episode psychosis and consisting of reduced attention and motivation, feelings of depression, anxiety, social withdrawal, suspiciousness, and most notably, sleep disorders.^12^

A growing body of evidence suggests that structural brain abnormalities and neural developmental alterations in the early stages of psychosis may lead to sleep disturbances and subsequent psychotic symptoms.^6^, ^13^, ^14^ The thalamus, a brain region implicated in both sleep regulation and psychosis, has been shown to exhibit early volume reduction in psychotic disorders, which worsens as the illness progresses.^15^ A study of individuals at ultra‐high risk for psychosis found that they had decreased bilateral thalamic volume and poorer sleep quality.^7^

Sleep deprivation also affects the risk of psychosis by impairing synaptic transmission.^16^, ^17^ Acetylcholine, involved in both sleep and hallucinations, has been proposed as a key factor. It is suggested that sleep deprivation could lead to hallucinations and the development of psychosis through cholinergic depletion.^16^, ^17^ A number of animal studies further indicate that sleep deprivation results in dopamine‐driven wakefulness and a consequent maladaptive hyperdopaminergic state, manifesting as paranoia and hallucinogenic behaviors.^18^, ^19^

An important interplay exists between dopamine, sleep disturbances, and psychosis. The dopamine hypothesis of schizophrenia postulates that hyperactivity in the dopaminergic system underlies schizophrenia symptoms. Specifically, an augmented density of D2 dopamine receptors and heightened receptor sensitivity can precipitate psychosis. Notably, D2 receptor agonists are known to bolster alertness while diminishing both slow‐wave and REM sleep,^20^ suggesting potential pathways linking dopaminergic system alterations with sleep disturbances and schizophrenia.

While a cross‐sectional study recorded one‐fourth of their study population experiencing first episode psychosis (FEP) with clinical insomnia,^9^ another study reported close to 80% of their study sample with early psychosis concurrently suffering from at least one sleep disorder, with insomnia and nightmare disorders as the most frequent.^1^ A large sample longitudinal analysis further identified that individuals with sleep disorders faced a doubled risk of both the onset and persistence of psychotic episodes.^3^

Disruptions in sleep patterns represent an important risk factor for psychosis that provides opportunities for preventative intervention. Both pharmacological and non‐pharmacological approaches can be utilized to positively influence sleep, thereby potentially mitigating psychosis onset or exacerbation.^13^, ^21^ Optimizing sleep quality stands out as a modifiable target that may help reduce vulnerability to psychotic experiences through medical and lifestyle management strategies. Cognitive behavioral therapy (CBT) for insomnia in the context of psychotic disorders has garnered significant clinical backing. Notably, CBT not only alleviates insomnia but also curtails symptoms like hallucinations and paranoia in controlled trials.^13^ Furthermore, specialized versions of CBT have been tailored expressly for psychotic conditions.

Basic sleep hygiene practices such as avoidance of caffeine or alcohol consumption, designation of the bedroom as only for sleep, maintenance of consistent sleep and wake times, and avoidance of daytime naps have been shown to be beneficial in people with psychosis.^21^ Improvement of sleep hygiene is particularly effective when paired with consumer‐grade devices like Fitbit. Such devices not only track sleep patterns and physical activity but also foster positive behavioral adjustments.^22^

Several pharmacological strategies exist for addressing sleep disturbances in psychosis. The most common medication has been antipsychotics. While antipsychotics demonstrate sedative properties that increase the sleep quantity patients receive, sleep quality does not necessarily improve.^23^ This finding suggests that antipsychotics treat the psychotic component of the illness more so than the sleep disturbance itself. Benzodiazepines can be used for short‐term management of insomnia; however, long‐term treatment in patients with schizophrenia has been associated with disrupted attention and decreased working memory.^24^ Orexin, FDA‐approved for primary insomnia since 2014, operates by potentially modifying dopaminergic firing and increasing dopamine release. While this mechanism suggests potential benefits for psychosis, extensive clinical studies are required to affirm its therapeutic relevance.^21^ Other proposed interventions, such as melatonin (N‐acetyl‐methoxytryptamine), bright light therapy, sleep restriction therapy, and more, have demonstrated promise in treating primary insomnia. Yet, their efficacy in managing insomnia associated with psychosis remains largely unexplored.^21^

## 6 CONCLUSION

Sleep disorders are strongly associated with the incidence and prevalence of psychotic disorders. This case report highlights the importance of evaluating sleep disturbances as a prelude to psychosis. In this case study, we discussed a previously stable patient with no prior psychiatric history who developed acute psychosis in the context of sleep disturbance. Given our observations, we advocate for comprehensive assessments of all patients exhibiting comorbid sleep and psychiatric symptoms to eliminate critical differentials, such as the emergence of first‐episode psychosis. Further research is needed to clarify the association between sleep disturbance and psychotic experiences and factors contributing to their interaction. The comparative effectiveness of antipsychotics and sleep‐specific therapeutic interventions also merits further investigation.

## AUTHOR CONTRIBUTIONS

**Jeffrey Kim:** Conceptualization; data curation; resources; writing – original draft. **Sakshi Prasad:** Data curation; methodology; writing – original draft; writing – review and editing. **Nikhita Suzanne Roshan:** Formal analysis; funding acquisition; investigation; validation; writing – original draft. **Bushra Farah Hasan:** Investigation; methodology; software; validation; writing – original draft. **Gurtej Gill:** Data curation; formal analysis; project administration; supervision; writing – original draft; writing – review and editing. **Sasidhar Gunturu:** Funding acquisition; investigation; methodology; project administration; supervision; validation; visualization; writing – original draft; writing – review and editing.

## FUNDING INFORMATION

The author(s) received no financial support for the research, authorship, and/or publication of this article.

## CONFLICT OF INTEREST STATEMENT

The author(s) declared no potential conflicts of interest with respect to the research, authorship, and/or publication of this article.

## ETHICS STATEMENT

Ethical approval was not needed with respect to a case report by our institution.

## CONSENT

Written informed consent was obtained from the patient for her anonymized information to be published in this article. The patient regained fair insight and judgment over the course of her treatment regime to provide written informed consent by herself.

## Data Availability

Data sharing not applicable–no new data generated.
